# New Patient Education Video on Colonoscopy Preparation: Development and Evaluation Study

**DOI:** 10.2196/15353

**Published:** 2020-10-21

**Authors:** Matthew T Bernstein, Jesse Garber, Patrick Faucher, Kristin A Reynolds, Gayle Restall, John R Walker, Harminder Singh

**Affiliations:** 1 Department of Psychology University of Manitoba Winnipeg, MB Canada; 2 IBD Clinical and Research Centre University of Manitoba Winnipeg, MB Canada; 3 George and Fay Yee Centre for Healthcare Innovation Winnipeg, MB Canada; 4 Department of Occupational Therapy University of Manitoba Winnipeg, MB Canada

**Keywords:** bowel preparation, evaluation study, medical informatics, information dissemination, information literacy, patient preference, implementation science, translational medical research, patient education

## Abstract

**Background:**

Although several patient education materials on colonoscopy preparation exist, few studies have evaluated or compared them; hence, there is no professional consensus on recommended content or media to use.

**Objective:**

This study aims to address this need by developing and evaluating a new video on colonoscopy preparation.

**Methods:**

We developed a new video explaining split-dose bowel preparation for colonoscopy. Of similar content videos on the internet (n=20), the most favorably reviewed video among patient and physician advisers was used as the comparator for the study. A total of 232 individuals attending gastroenterology or urology clinics reviewed the new and comparator videos. The order of administration of the new and comparator videos was randomly counterbalanced to assess the impact of presentation order. Respondents rated each video on the following dimensions: information amount, clarity, trustworthiness, understandability, new or familiar information, reassurance, information learned, understanding from the patient’s point of view, appeal, and the likelihood of recommending the video to others.

**Results:**

Overall, 71.6% (166/232) of the participants preferred the new video, 25.0% (58/232) preferred the comparator video, and 3.4% (8/232) were not sure. Furthermore, 64.0% (71/111) of those who viewed the new video first preferred it, whereas 77.7% (94/121) of the participants who viewed the new video second preferred it. Multivariable logistic regression analysis also demonstrated that participants were more likely to prefer the new video if they had viewed it second. Participants who preferred the new video rated it as clearer and more trustworthy than those who preferred the comparator video.

**Conclusions:**

This study developed and assessed the strengths of a newly developed colonoscopy educational video.

## Introduction

### Background

The lifetime risk of developing colorectal cancer is 4.5% among men and 4.2% among women in the United States [1]. The chief defense against colorectal cancer morbidity and mortality is through prevention and early detection by screening for colorectal cancer and precursor colorectal polyps. Colonoscopy is essential as the first-line colorectal cancer screening test, to follow up on the positive results of other initial colorectal cancer screening tests, for surveillance of those with colorectal neoplasia, and for assessment of symptoms such as rectal bleeding. An accurate and successful colonoscopy involves an onerous patient preparation, including cleansing the colon of residual materials. However, 10% to 20% of colonoscopies continue to fail because of poor preparation [2]. Poor preparation can lead to increased duration and repetition of the colonoscopy [3], which, in addition to recipient inconvenience and worse health care outcomes, leads to increased costs [4]. Educational materials such as videos can improve bowel preparation and may reduce the need for repeat colonoscopy [5,6].

Colonoscopy is an invasive test, and there can often be a considerable amount of anxiety associated with this procedure [7]. One way to mitigate this anxiety is by providing patients with information [8]. Much of the existing information is available in written format. Our research team recently published a study evaluating revised written colonoscopy materials that were found to be superior to existing written materials [9]. However, past research by a related group found that people are interested in health information delivered in a variety of formats, including written and video formats [10-12]. In fact, clinicians use a range of different materials to inform patients about colonoscopy preparation [13]. Nevertheless, it is not well understood how patients perceive such information, although enhanced instructions improve the quality of bowel preparation [14]. Previous research indicates that patients and their families have several questions about colonoscopy that are not fully answered by existing resources [15-17]. Moreover, although several educational videos on colonoscopy are available, most of them have not been evaluated systematically. Prior studies have often not asked participants about their information needs or their assessment of the quality of the information provided in the videos.

Most importantly, there are no previous studies that have comparatively evaluated different educational videos. Therefore, physicians and clinical practice groups have little information to guide them in the selection of enhanced educational materials, including educational videos. As such, current guidelines do not recommend specific enhanced instruction materials for colonoscopy preparation, in either written or video format [15].

### Information Quality

The field of social psychology has paved the way for evaluation studies examining participants’ responses to two or more targets (eg, photos or information about people seen in social settings). However, very little research has been conducted by applying this methodology to compare different health-related resources such as videos. Arazy et al [18] developed an approach to evaluate information quality using heuristic principles of a multidimensional construct including dimensions such as accuracy, completeness, objectivity, and representation. Furthermore, it is important for patients to understand the health information presented to them. Nguyen and Wieland [19] suggested that low health literacy may lead to inadequate preparation, which underscores the importance of making information accessible to people of all backgrounds. Educational videos are advantageous as they may be more accessible to those with low health literacy [20]. The methodology presented in this study allows for an evaluation of the information quality of 2 colonoscopy videos and for clearer judgments about how different resources compare with each other.

### Order Effects

Murdock [21] published an influential paper describing the U-shaped serial position curve that depicts the order effects of recall occurring in short-term memory. He went on to explain that primacy effects represent better memory for stimuli presented first, recency effects represent better memory for stimuli presented last, and worst memory occurs for stimuli presented in between, which produces a U-shaped curve. Indeed, people prefer to recall information in forward serial order even when it is not required by the task [22-24]. Most research in this area has focused on the recall of numbers, letters, and words; very little research has been done on the order effects of larger quantities of information, including video clips. We have begun to fill this gap with a recent study examining the order effects of rating colonoscopy information sheets [9]. In that study, we demonstrated a clear order effect for our revised information sheet: a greater preference if it was viewed first.

This research builds on existing research evaluating patient-oriented educational videos by having the same individuals compare 2 videos directly. As the goal of a new video is to be an improvement over currently available videos, we were interested in how the new video compared with an existing high-quality video in terms of quality and patient preference. The new video assessed in this study was developed by our research team. At the time of video development, we were unable to identify a video that clearly described the split-dose method of bowel preparation. The new video was conceptualized to address this content gap, as split-dose bowel preparation has been shown to lead to superior bowel cleansing and higher colonic polyp detection rates [25,26]. Split-dose bowel preparation involves the intake of half of the preparation laxative on the day before colonoscopy and half on the day of the colonoscopy.

## Methods

### Overview

In 2017, our research team developed a project titled “Optimizing colonoscopy procedures and reducing unnecessary and over use” and explored the information needs and preferences of patients undergoing colonoscopy [16]. On the basis of the expressed needs and inputs from patients and health care providers, we developed revised educational resources for patients considering or preparing for colonoscopy. The educational materials went beyond simply explaining the preparation instructions and used visual aids and clear language with less medical jargon, shorter sentences, and brief paragraphs, with the goal of making the information clearer to the average reader [27,28]. Given that visual explanations may enhance learning [29], we also developed videos to demonstrate a patient’s experience of preparing for and undergoing a colonoscopy. These and other educational materials developed by our research team (including videos) can be accessed at MyColonoscopy [30]. The written materials have Creative Commons licenses; therefore, they may be used in other settings.

### Video Selection

#### New Video Development

To supplement the written materials available on the MyColonoscopy website, our team developed 2 colonoscopy educational videos. The content for the step-by-step patient education video on preparation was informed by a study that included the development of a novel patient educational booklet [31]. A recent review [32] of web-based colonoscopy bowel preparation videos further assisted efforts to identify key content areas to address in the videos. Finally, an expert advisory group and interviews with individuals who had recently undergone colonoscopy provided additional insights on the barriers to good quality colonoscopy preparation [33]. Feedback from advisory groups ultimately led to a much stronger final product. As our research team is located in Canada, versions of this video are available in both English and French. We developed videos for colonoscopy preparation and patient experience for colonoscopy. In this study, we evaluated the English version of the video on preparing for colonoscopy (the video is 6 minutes long).

#### Comparison of Video Selection

The comparator video was selected by searching YouTube with terms such as “colonoscopy preparation,” “preparing for colonoscopy,” “colonoscopy prep,” and “bowel prep.” The results yielded several (n=20) videos that varied in length (some were too long compared with the length of the new video), varied in the amount of information provided on the process of bowel preparation, and did not involve a demonstration of the preparation. Ultimately, we narrowed it down to 3 videos that were relatively short (under 10 min) and focused on the bowel preparation aspect of colonoscopy. We then surveyed the expert advisory (individuals who had not participated in the development of the revised video) and patient advisory groups (mentioned above) by asking them to rate each video on the following dimensions: amount of information, clarity, trustworthiness, ease of viewing and understanding, novelty or familiarity of the information (very familiar to very new), reassurance (very worried to very reassured), information learned, understanding from the patient’s point of view, appeal, and whether they would recommend the video to someone undergoing colonoscopy. The highest-ranked video was from the University of Utah Healthcare and made for a strong comparison with our new video (the video is 3 minutes long). Reference to the video developer or originating site was removed from both videos for evaluation purposes.

### Participants

Participants were recruited from the waiting rooms of gastroenterology and urology clinics at the 2 largest hospitals and 2 community-based outpatient gastroenterology clinics in Winnipeg, Manitoba. The patients were seen in this setting for consultation on a wide variety of gastrointestinal and urological problems. Participants were invited by a research assistant to complete a survey evaluating the 2 colonoscopy videos. If the person agreed, the research assistant provided them with an information sheet with a brief description of the study and a web address to complete the survey on the web. They could complete the web-based survey at their convenience. A total of 3 different recruitment approaches were used to recruit participants from the clinics. First, some participants were given a Can $10 (US $7.47) gift card when they agreed to participate but before the completion of the survey; this had a response rate of 46.0% (127 of 276 who were approached to participate completed the survey). Second, some participants received a gift card after completing the survey; this had a response rate of 43.8% (77/176). A final recruitment approach involved emailing invitations to participants who had completed previous survey studies by our group. This group received a gift card after completion (response rate of 47%, 28/60). The overall response rate was 45.3% (232/512). All participants reviewed the videos independently after they left the clinics.

### Measurement

Participants were asked to review one at a time the new and comparator videos, where the order of video presentation was randomly counterbalanced. They were then asked to rate each video on the following dimensions: amount of information, clarity, trustworthiness, ease of viewing and understanding, novelty or familiarity of the information (very familiar to very new), reassurance (very worried to very reassured), information learned, understanding from the patient’s point of view, appeal, and whether they would recommend the video to someone undergoing a colonoscopy. These dimensions were rated using a 5-point Likert-type scale. Open-ended questions included likes and dislikes about the videos and suggestions for improvement. After the participants viewed both videos and responded to these questions, they were asked, “Which video do you think would be most helpful for people who are considering having a colonoscopy?” They were then asked 4 comparison questions on similar dimensions to those described above (ie, clarity, trustworthiness, ease of watching and understandability, and reassurance). Finally, they were asked an open-ended question about why their preferred video was better than the other video. Participants were also asked background questions, including age, sex, primary language spoken, education, history of gastroenterology visits, and history of a colonoscopy. The survey questions used in this study are given in Multimedia Appendix 1. Multimedia Appendices 2 and 3 contain the new and comparator videos, respectively. This study was approved by the University of Manitoba Health Research Ethics Board.

### Statistical Methods

IBM SPSS statistics version 24.0 was used to conduct the data analysis. Descriptive statistics (including means and proportions) were used to summarize sociodemographic information and the responses to questions about video ratings and preferences. Confidence intervals are reported as they are typically used in survey research and allow for convenient comparisons within and across different survey questions and groups of respondents. Confidence intervals have been recommended rather than pairwise significance tests for this type of comparison as they help the reader understand the magnitude of differences rather than simply concluding whether a difference is statistically significant [34,35].

Logistic regression was used to examine the predictors of preference for the new video. The following predictors were used: order, previous colonoscopy, gender, age, education, and language most often spoken at home. A median-split approach was used to transform age and education into dichotomous variables.

Open-ended questions were analyzed using a descriptive content analysis approach [36]. Authors MB and JG coded these responses and organized codes into categories.

## Results

As can be seen in Table 1, the group viewing the new video first was very similar to the group viewing the comparator video first. More than half of each sample was female and had a little over three years of education after high school. Most of each sample had previously seen a gastroenterologist and undergone a colonoscopy.

**Table 1:** Sociodemographic characteristics of respondents.

Characteristics	New video first (n=111)	Comparator video first (n=121)
Age (years), mean (95% CI)	52.21 (49.10-55.32)	52.16 (49.61-54.71)
Female, % (95% CI)	52.3 (42.6-61.8)	57.9 (48.5-66.8)
English as primary language, % (95% CI)	92.8 (86.3-96.8)	92.6 (86.3-96.5)
Education (years), mean (95% CI)	15.54 (14.81-16.27)	15.99 (15.26-16.72)
Had visited a gastroenterologist before the current colonoscopy, % (95% CI)	64.0 (54.3-72.9)	71.1 (62.1-79.0)
Had a previous colonoscopy, % (95% CI)	68.5 (59.0-77.0)	59.5% (50.2-68.3)

### Video Preference

Overall, 71.6% (166/232) of the participants preferred the new video, 25.0% (58/232) preferred the comparator video, and 3.4% (8/232) were not sure. Table 2 displays the results for participants’ preferred video based on the order of presentation. Almost two-thirds of those who viewed the new video first preferred it. Interestingly, more than three-quarters of those who viewed the comparator video first preferred the new video, and there was a larger difference in preference for the new video in this group. Table 3 displays the results for participants’ preferred video based on the history of colonoscopy. Almost three-quarters of individuals who had previously received a colonoscopy preferred the new video, whereas two-thirds of those who had not previously received a colonoscopy preferred the new video, with overlapping confidence intervals.

**Table 2:** Preferred video related to the order of presentation of the videos.

Preference	New video first (n=111)		Comparator video first (n=121)	
	Participants, n (%)	95% CI	Participants, % (n)	95% CI
Prefer new video	71 (64.0)	54.3-72.9	94 (77.7)	69.2-84.8
Prefer comparator video	40 (30.6)	22.2-40.1	27 (19.8)	13.1-28.1
Difference in preference, %	33.4	24.7-42.9	57.9	48.5-66.8

**Table 3:** Preferred video related to history of colonoscopy.

Preference	Previous colonoscopy (n=148)		No previous colonoscopy (n=84)		
	Participants, n (%)	95% CI		Participants, n (%)	95% CI
Prefer new video	109 (74.3)	66.5-81.1		55 (66)	54-76
Prefer comparator video	39 (23.0)	16.5-30.6		29 (29)	19-40
Difference in preference, %	51.3	43.0-59.6		37	27-48

Table 4 examines the predictors of preference for the new video. Of the 6 potential predictors, only 1 was significant. Participants were twice as likely to prefer the new video if they had viewed the comparator video first, which is consistent with the results from Table 2 (odds ratio 2.20, 95% CI 1.16-4.18).

**Table 4:** Predictors of preference for new video (n=232).

Predictor	Odds ratio (95% CI)
Order (0^a^=new video first and 1=comparator video first)	2.20^b^ (1.16-4.18)
Previous colonoscopy (0^a^=yes and 1=no)	1.31 (0.68-2.54)
Gender (0^a^=male and 1=female)	1.43 (0.76-2.71)
Age (0^a^=aged <55 years and 1=aged ≥55 years)	1.84 (0.97-3.49)
Education (0^a^=<16 years and 1=≥16 years)	0.78 (0.41-1.49)
Language spoken at home (0^a^=not English and 1=English)	1.20 (0.38-3.75)

^a^0 corresponds to the reference group.

^b^Significance is present if confidence interval does not cross 1.

### Video Ratings

Multimedia Appendix 4 displays the overall mean ratings of the evaluated dimensions of each video regardless of the order or previous colonoscopy experience. As can be seen, the new video received higher ratings in all categories except familiarity compared with the comparator video.

Table 5 displays the components of evaluation ratings of the 2 videos by colonoscopy experience (one or more previous colonoscopies vs no previous colonoscopy). It was found that the new video received higher evaluation ratings in almost all categories, regardless of previous colonoscopy experience. Among those who had previously undergone colonoscopy, the new video received significantly higher ratings in every category except trustworthiness. Among those with no prior colonoscopy experience, the new video received significantly higher ratings on the amount of information, understanding from the patient’s perspective, video appeal, and whether they would recommend the video. Not surprisingly, individuals who previously had a colonoscopy rated the content of both videos as more familiar.

**Table 5:** Rating of the dimensions of the current and revised videos, stratified by previous colonoscopy experience.

Dimension	Rating of new video (n=232), mean (95% CI)		Rating of comparator video (n=232), mean (95% CI)	
	Had colonoscopy previously (n=148)	No previous colonoscopy (n=84)	Had colonoscopy previously (n=148)	No previous colonoscopy (n=84)
Amount of information^a^	3.07^b^ (3.01-3.13)	3.12^c^ (3.03-3.21)	2.82 (2.73-2.92)	2.89 (2.80-2.99)
Clarity^d^	4.31^b^ (4.18-4.44)	4.37 (4.26-4.48)	3.75 (3.60-3.90)	4.13 (3.98-4.28)
Trustworthy^d^	4.28 (4.15-4.41)	4.30 (4.16-4.44)	4.01 (3.98-4.26)	4.12 (3.98-4.26)
Easy to watch or understand^d^	4.37^b^ (4.26-4.48)	4.27 (4.15-4.40)	3.90 (3.76-4.04)	4.13 (3.97-4.29)
Familiarity^e^	1.95^b^ (1.78-2.12)	3.41 (3.12-3.68)	2.10 (1.93-2.26)	3.49 (3.24-3.74)
Reassurance^f^	3.85^b^ (3.72-3.97)	3.73 (3.53-3.92)	3.46 (3.32-3.60)	3.55 (3.38-3.71)
Information learned^d^	4.01^b^ (3.87-4.14)	4.14 (4.00-4.28)	3.68 (3.54-3.81)	3.86 (3.67-4.05)
Understand patient’s point of view^g^	3.91^b^ (3.76-4.06)	3.95^c^ (3.79-4.11)	3.30 (3.13-3.47)	3.54 (3.31-3.76)
Appealing^d^	3.98^b^ (3.85-4.11)	3.93^c^ (3.76-4.09)	3.39 (3.24-3.54)	3.52 (3.35-3.70)
Recommend video^d^	4.28^b^ (4.17-4.39)	4.23^c^ (4.09-4.36)	3.70 (3.54-3.85)	3.83 (3.65-4.02)

^a^The amount of information was rated on a scale from 1 (too little) to 5 (way too much).

^b^These values denote nonoverlapping confidence intervals in the previous colonoscopy group; comparison of new versus comparator video.

^c^These values denote nonoverlapping confidence intervals in the no previous colonoscopy group; comparison of new versus comparator video.

^d^All other variables were rated on a scale from 1 (strongly disagree) to 5 (strongly agree).

^e^The familiarity variable was rated on a scale from 1 (very familiar) to 5 (very new).

^f^Reassurances were rated on a scale from 1 (very worried) to 5 (very reassured).

^g^Understand patient’s point of view (understanding what it is like to have a colonoscopy from the patient’s point of view).

Table 6 displays the components of the evaluation ratings of the 2 videos according to the order of presentation of the videos. It was found that the new video received higher evaluation ratings regardless of the viewing order. Participants who viewed the new video first provided higher ratings to it in every category except trustworthiness and familiarity. If the comparator video was viewed first, it did not obtain any ratings higher than the new video.

**Table 6:** Evaluation of the dimensions of the current and revised videos, stratified by the order of presentation of videos.

Dimension	New video viewed first, mean (95% CI)		Comparator video viewed first, mean (95% CI)	
	New video (n=111)	Comparator video (n=111)	New video (n=121)	Comparator video (n=121)
Amount of information^a^	3.05 (2.99-3.12)	2.67 (2.55-2.78)	3.12 (3.04-3.19)	3.02 (2.95-3.08)
Clarity^b^	4.28^c^ (4.14-4.42)	3.66 (3.51-3.86)	4.38 (4.26-4.50)	4.07 (3.93-4.22)
Trustworthy^b^	4.19 (4.03-4.35)	3.96 (3.82-4.09)	4.37 (4.26-4.48)	4.14 (4.00-4.28)
Easy to watch or understand^b^	4.31^c^ (4.19-4.42)	3.78 (3.62-3.93)	4.36 (4.25-4.48)	4.17 (4.03-4.31)
Familiarity^d^	2.36 (2.12-2.60)	2.51 (2.29-2.74)	2.58 (2.33-2.83)	2.68 (2.44-2.91)
Reassurance^e^	3.69^c^ (3.55-3.82)	3.34 (3.19-3.50)	3.91 (3.75-4.07)	3.63 (3.49-3.77)
Information learned^b^	4.08^c^ (3.96-4.20)	3.48 (3.32-3.64)	4.03 (3.87-4.19)	3.98 (3.84-4.12)
Understand patient’s point of view^f^	3.75^c^(3.59-3.91)	3.32 (3.14-3.51)	4.09 (3.94-4.25)	3.44 (3.24-3.64)
Appealing^b^	3.78^c^ (3.64-3.91)	3.29 (3.10-3.47)	4.13 (3.98-4.28)	3.58 (3.44-3.72)
Recommend video^b^	4.16^c^ (4.05-4.27)	3.49 (3.30-3.68)	4.35 (4.22-4.48)	3.98 (3.84-4.12)

^a^The amount of information was rated on a scale from 1 (too little) to 5 (way too much).

^b^All other variables were rated on a scale from 1 (strongly disagree) to 5 (strongly agree).

^c^Denotes nonoverlapping confidence intervals in the group that viewed the new video first.

^d^The familiarity variable was rated on a scale from 1 (very familiar) to 5 (very new).

^e^Reassurances were rated on a scale from 1 (very worried) to 5 (very reassured).

^f^Understand patient’s point of view (understanding what it is like to have a colonoscopy from the patient’s point of view).

By comparing with the 2 videos (Table 7), it was found that participants who preferred the new video gave higher clarity and trustworthiness ratings than those who preferred the comparator video.

**Table 7:** Comparison ratings of videos, stratified by preferred video.

Comparison dimension	Preferred new video (n=165), mean (95% CI)	Preferred comparator video (n=58), mean (95% CI)
Clarity compared with the other video^a^	3.42^b^ (3.30-3.54)	2.79^b^ (2.58-3.01)
Trustworthiness compared with the other video^c^	2.89^b^ (2.76-3.02)	2.38^b^ (2.20-2.56)
Readability or understandability compared with the other video^d^	3.21 (3.08-3.34)	2.91 (2.71-3.12)
Reassurance compared with the other video^e^	3.09 (2.97-3.22)	2.81 (2.62-3.00)

^a^Rating scale for clarity is from 1 (less clear than the video I did not prefer) to 4 (clearer than the video I did not prefer).

^b^Denotes nonoverlapping confidence intervals.

^c^Rating scale for trustworthiness is from 1 (less trustworthy than the video I did not prefer) to 4 (more trustworthy than the video I did not prefer).

^d^Rating scale for readability is from 1 (less easy to read and understand than video I did not prefer) to 4 (easier to read and understand than video I did not prefer).

^e^Rating scale for reassurance is from 1 (more worrying than the video I did not prefer) to 4 (more reassuring than the video I did not prefer).

Multimedia Appendix 5 displays the components of evaluation ratings of the 2 videos by education level (low education=less than 16 years vs high education=16 years or more). It was found that the new video received higher evaluation ratings regardless of the education level. Those in the lower education level group rated the new video more favorably than the comparator video in almost all dimensions (other than *familiarity*).

Multimedia Appendix 6 includes the Pearson correlations of the variables used to evaluate the 2 videos. Cohen [37] suggested cutoff scores for small (*r*=.1), medium (*r*=.3), and large (*r*=.5) Pearson correlations. For the new video, almost all the correlations were significant at the .01 level. For instance, there were moderate and significant positive correlations for clarity and trustworthiness, ease of watching or understandability, reassurance, patient’s point of view, appeal, and likelihood of recommending the video to others. Familiarity was not related to most variables; however, it was positively associated with the information learned. A very similar pattern was observed for the ratings of the comparator video. The small-to-moderate size of most correlations suggest that the concepts are related but not completely overlapping. Appeal and recommendation of the video to others had moderate-to-large positive correlations in most categories, suggesting that these 2 (appeal and recommendation to others) are summarizing variables.

In the content analysis of open-ended questions, we found that many participants liked the amount of information or details in the new video, whereas others felt that the new video contained too much information and thought that the video was too long. The amount of information in the new video was identified as a strength among those who preferred it. On the other hand, the shorter video length was identified as a strength among those who preferred the comparator video. Many participants commented on the pacing of the video and found the narration in the new video to be easier to listen to and follow; many felt that the pace of the comparator video was too fast. Regardless of the video, clarity of the information was important to viewers. Visuals and graphics within the video were important for a few respondents. A few participants indicated that they would have liked information about *red flags* (ie, things that could go wrong) related to the preparation.

## Discussion

### Principal Findings

We have developed and assessed patient preferences for a new colonoscopy educational video. This is one of the first studies to evaluate revised educational materials and directly compare them with existing materials among a group of participants (a within-subject design). It builds on a recently published study by our group [9] that outlined a novel methodological approach for evaluating consumers’ judgments concerning the quality of newly developed written colonoscopy information in comparison with existing written information.

An order effect was demonstrated in the previous study and in this study. Participants were twice as likely to prefer the new video if they had viewed the comparator video first—the recency effect; that is, if the new video were viewed second, it was more strongly preferred. These findings emphasize the importance of counterbalancing in a comparative design study to ensure that order effects are assessed and accounted for. There has been little previous research done in this area, particularly regarding the evaluation of health information, including patient colonoscopy preparation educational materials.

In this study, we also examined whether there was a difference in response rate using different recruitment methods. A similar response rate among different recruitment methods suggested that the participants were unlikely to complete the survey if they were provided a gift card before the completion of the survey. It will be more efficient (and economical) in future studies to provide a gift card after the completion of the survey.

We are not aware of any previous study that compared 2 colonoscopy educational videos in a randomized controlled trial (RCT). An RCT compared standard written colonoscopy preparation instructions with written instructions and a video that provided visual instructions about the preparation process [6]. Patients randomized to the video condition had better ratings of bowel preparation than those in the standard instructions condition, but there was no difference in satisfaction with the procedure.

In our study, almost three-quarters of individuals who had previously undergone a colonoscopy preferred the new video. This is probably because of the fact that people who have previously undergone colonoscopy have a better understanding of the effort and steps required to prepare and undergo the procedure compared with those who have not. However, most participants, regardless of their colonoscopy experience, still preferred the new video. Regardless of previous colonoscopy experience, colonoscopy procedure can still cause significant anxiety in patients. Providing higher quality educational materials can help alleviate some of this anxiety [8]. The new video helped viewers feel more reassured compared with the comparator video, which is important in alleviating some of this anxiety.

Given that many patients do not feel adequately informed about the colonoscopy procedure [16], it is crucial to provide patients with materials to enhance their understanding. In a recent systematic review of enhanced education for bowel preparation (ie, counseling or training sessions, educational booklets, or videos), researchers found that enhanced education methods improve bowel preparation and promote better visualization of the colon in patients preparing for colonoscopy [38]. The advantages of some of these approaches are that they are widely accessible and cost less [38]. A video may have an additional advantage of being comprehensible to people of varying levels of health literacy [20]. Previous research has demonstrated that the comprehension of colonoscopy information is one factor that is related to health literacy and suggests the importance of developing materials for individuals with varying levels of health literacy [39]. In this study, participants with lower levels of education rated the new video more favorably than the comparator video in almost all dimensions, which suggests an enhanced role of the new video in clinical practice. On the other hand, written materials have an advantage as patients can review specific sections of information that are of interest to them. Therefore, presenting information in different formats provides consumers the options to select their preferred format for obtaining information.

### Limitations

This study has a few limitations. First, most participants were enrolled in this study through waiting room recruitment; therefore, whether the results would be generalizable to those coming directly for colonoscopy will need to be evaluated in future studies. Although the overall response rate was reasonable (232/512, 45.3%), we cannot comment on the perceptions of nonrespondents, as in any other survey study. The survey included a reasonable number of people (84/232, 36.2%) with no previous experience with colonoscopy. The survey included mainly older adults and had a limited number of people who were younger, did not speak English at home, and had very limited education. This may limit the generalizability of the findings to these other groups.

### Conclusions

We have developed a new colonoscopy educational video based on the reported needs of patients and health care providers and demonstrated patients’ preference for this new resource as compared with a high-quality video. We have also developed an approach to evaluate and compare different educational materials, which yielded a preference for the new educational video. This study extended our previous findings with counterbalanced presentation of information [9], demonstrating an order effect in evaluation studies. This approach can be used to evaluate other patient-centered information materials. The next step in this research would be to determine whether the new (patient-preferred) video has a better impact than a comparator video on the quality of the bowel preparation and/or leads to more successful colonoscopy and assessment among different patient populations. Other future directions include determining (1) the effectiveness of video education for colonoscopy among very low-literacy populations and among populations who have historically poor preparation rates and (2) whether providing a *good* educational video on the web increases the likelihood of primary care practitioners providing information on bowel preparation for colonoscopy to patients and/or encourages patients to ask for the split-dose method of bowel preparation (more efficacious but involving early morning awakening). A final area of future study should be the effects of video length and presentation on its effectiveness (eg, diversity of the person in the video and/or whether trustworthiness is increased if a physician is profiled rather than a patient).

## Appendix

Multimedia Appendix 1 Survey questions.

Multimedia Appendix 2 New video.

Multimedia Appendix 3 Comparator video.

Multimedia Appendix 4 Overall ratings.

Multimedia Appendix 5 Table with ratings stratified by education.

Multimedia Appendix 6 Correlations.

## Abbreviations

RCT: randomized controlled trial
