# Supplementary material for: New Patient Education Video on Colonoscopy Preparation: Development and Evaluation Study
Source: JMIR Hum Factors. 2020 Oct 21;7(4):e15353. doi: 10.2196/15353 (PMC7641787; doi:10.2196/15353)
Supplement: Multimedia Appendix 1 [file humanfactors_v7i4e15353_app1.docx]

**PATIENT INFORMATION SURVEY** [BP form uv]

**Colonoscopy Information**

**Bowel Preparation Instructions**

Our team is evaluating educational materials developed for the public about common medical procedures and preparation instructions for these medical procedures. We would appreciate your opinion about two different versions of some of this material. We are interested in which version you think may be more helpful for people considering colonoscopy. If you are scheduled for a colonoscopy the instructions you receive in the video are coordinated with the written material you receive from the colonoscopy centre.

**COLONOSCOPY:** When a patient sees a doctor specialized in gastroenterology or a family doctor, it is sometimes recommended that they have this test. A colonoscopy is a procedure which allows the inside of the colon (also called large intestine or large bowel) to be examined using a long thin flexible tube with a tiny video camera at the tip. Colonoscopies help doctors diagnose probable causes of rectal bleeding, diarrhea and sometimes, chronic abdominal pain. Colonoscopy is used to look for colon polyps and early signs of colon cancer.

Please provide your opinions by reviewing Video X and Video Y of this information about colonoscopy and answering the questions. Questions later in the survey will ask you to indicate which version you prefer. You may look back and forth between the two videos in making these judgments.

**Video X: Please press the play button to start the video.**

**Your opinion:** Considering a person who was going to have a colonoscopy and would like information about this aspect of colonoscopy.

U1. **The amount of information on this video was:**

( ) much too little

( ) too little

( ) just right

( ) too much

( ) way too much

U2. **The information in this video is clear.**

( ) strongly disagree

( ) disagree

( ) neutral

( ) agree

( ) strongly agree

U3. **The information in this video seems trustworthy.**

( ) strongly disagree

( ) disagree

( ) neutral

( ) agree

( ) strongly agree

U4. **The information in this video is easy to watch and understand.**

( ) strongly disagree

( ) disagree

( ) neutral

( ) agree

( ) strongly agree

U5. **How familiar to you or new to you is the information in this video?**

( ) very familiar

( ) familiar

( ) unsure

( ) new

( ) very new

U6. **This video would leave someone who will be having a colonoscopy feeling**

O very worried

O worried

O neutral

O reassured

O very reassured

U7. **I learned a lot of information about preparing for a colonoscopy in this video.**

( ) strongly disagree

( ) disagree

( ) neutral

( ) agree

( ) strongly agree

U8. **The video helped me understand what it is like to have a colonoscopy from the patient’s point of view.**

( ) strongly disagree

( ) disagree

( ) neutral

( ) agree

( ) strongly agree

U9. **This video appealed to me.**

( ) strongly disagree

( ) disagree

( ) neutral

( ) agree

( ) strongly agree

U10. **I would recommend this video for those undergoing colonoscopy.**

( ) strongly disagree

( ) disagree

( ) neutral

( ) agree

( ) strongly agree

U7. **What did you like about the material?**

U8. **What did you dislike about the material?**

U9. **Do you have any suggestions for improving the material or for other things that should be included?**

**Video Y: Please press the play button to start the video.**

**Your opinion:** Considering a person who was going to have a colonoscopy and would like information about this aspect of colonoscopy.

U1. **The amount of information on this video was:**

( ) much too little

( ) too little

( ) just right

( ) too much

( ) way too much

U2. **The information in this video is clear.**

( ) strongly disagree

( ) disagree

( ) neutral

( ) agree

( ) strongly agree

U3. **The information in this video seems trustworthy.**

( ) strongly disagree

( ) disagree

( ) neutral

( ) agree

( ) strongly agree

U4. **The information in this video is easy to watch and understand.**

( ) strongly disagree

( ) disagree

( ) neutral

( ) agree

( ) strongly agree

U5. **How familiar to you or new to you is the information in this video?**

( ) very familiar

( ) familiar

( ) unsure

( ) new

( ) very new

U6. **This video would leave someone who will be having a colonoscopy feeling**

O very worried

O worried

O neutral

O reassured

O very reassured

U7. **I learned a lot of information about preparing for a colonoscopy in this video.**

( ) strongly disagree

( ) disagree

( ) neutral

( ) agree

( ) strongly agree

U8. **The video helped me understand what it is like to have a colonoscopy from the patient’s point of view.**

( ) strongly disagree

( ) disagree

( ) neutral

( ) agree

( ) strongly agree

U9. **This video appealed to me.**

( ) strongly disagree

( ) disagree

( ) neutral

( ) agree

( ) strongly agree

U10. **I would recommend this video for those undergoing colonoscopy.**

( ) strongly disagree

( ) disagree

( ) neutral

( ) agree

( ) strongly agree

V7. **What did you like about the material?**

V8. **What did you dislike about the material?**

V9. **Do you have any suggestions for improving the material or for other things that should be included?**

**Comparing the first and second video:**

S1. **Which video do you think would be most helpful for people who are preparing for a colonoscopy?**

O The first video I watched

O The second video I watched

O Not sure [SKIP TO THE BACKGROUND QUESTIONS SECTION BELOW, QUESTION S7]

S2. **Comparing your preferred video with the other video, the preferred video is:**

O less **clear** than the video I did not prefer

O about as **clear** as the video I did not prefer

O somewhat more **clear** than the video I did not prefer

O much more **clear** than the video I did not prefer

S3. **Comparing your preferred video with the other video, the preferred video is:**

O less **trustworthy** than the video I did not prefer

O is about as **trustworthy** as the video I did not prefer

O is a somewhat more **trustworthy** than the video I did not prefer

O is much more **trustworthy** than the video I did not prefer

S4. **Comparing your preferred video with the other video, the preferred video is:**

O less easy **to watch and understand** than the video I did not prefer

O about the same **to watch and understand** as the video I did not prefer

O somewhat easier **to watch and understand** than the video I did not prefer

O much easier **to watch and understand** than the video I did not prefer

S5. **Comparing your preferred video with the other video, the preferred video is:**

O more **worrying** than the video I did not prefer

O about the **same in reassurance** as the video I did not prefer

O somewhat more **reassuring** than the video I did not prefer

O much more **reassuring** than the video I did not prefer

S6. **Why do you think your preferred video is better?**

**Background questions.**

This section asks a few questions about you.

S7. **Have you seen a specialist in gastroenterology before?**

O No

O Yes If yes about how many times: ______

S8. **Have you ever had a colonoscopy?**

O No

O Yes **If yes about how many times**: ______

S9. **IF YES, when did you have the last colonoscopy?**

O Within the past year

O One to two years ago

O More than 2 years but less or equal to 5 years ago

O More than 5 years but less or equal to 10 years ago

O More than 10 years ago

S10. **Your sex:** O Male O Female O Prefer not to answer

S12. **Your age:** ______

**How many years of education have you completed in the following areas?**

S13. **Completed high school diploma:** O Yes O No

S14. **Apprenticeship:** O 0 O 1 O 2 O 3 O 4 O 5 years

S15. **College, technical, business, vocational, nursing (non-university):**

O 0 O 1 O 2 O 3 O 4 O 5 O 6 years

S16. **University program**:

O 0 O 1 O 2 O 3 O 4 O 5 O 6 O 7 O 8 O 9 O 10 O 11 O 12 or more

S17. **What is the language you most often speak at home?**

O English

O French

O Another language **What language?** ________________

S18. **Do you have?**

O Crohn’s disease

O Ulcerative colitis

O Irritable bowel syndrome

O Family history of colon cancer

O Other stomach/belly condition – Please specify: ______________________________

S19. **Your postal code:** **__ __ __ __ __ __**

**Thank you for completing the survey. Your opinions will be very helpful in evaluating the videos and to improve the information available to people when they are preparing for a colonoscopy!**
