# Supplementary material for: New Patient Education Video on Colonoscopy Preparation: Development and Evaluation Study
Source: JMIR Hum Factors. 2020 Oct 21;7(4):e15353. doi: 10.2196/15353 (PMC7641787; doi:10.2196/15353)
Supplement: Multimedia Appendix 4 [file humanfactors_v7i4e15353_app4.docx]

Appendix 4 Overall rating of Characteristics of Comparator and New Videos

|  | Mean (95%CI) | |
| --- | --- | --- |
|  | New Video  (N=232) | Comparator Video  (N=232) |
| Amount of Information (1-5) | **3.09**  **(3.03, 3.14)** | 2.85  (2.78, 2.92) |
| Clarity (1-5) | **4.33**  **(4.24, 4.42)** | 3.89  (3.77, 4.00) |
| Trustworthy (1-5) | **4.28**  **(4.19, 4.38)** | 4.05  (3.96, 4.15) |
| Easy to Watch/Understand (1-5) | **4.34**  **(4.25, 4.42)** | 3.98  (3.88, 4.09) |
| Familiarity (1-5) | 2.47  (2.30, 2.65) | 2.60  (2.44, 2.76) |
| Reassurance (1-5) | **3.80**  **(3.70, 3.91)** | 3.49  (3.39, 3.60) |
| Information Learned (1-5) | **4.06**  **(3.96, 4.16)** | 3.74  (3.63, 3.85) |
| Understand Patient’s POV (1-5) | **3.93**  **(3.81, 4.04)** | 3.38  (3.25, 3.52) |
| Appealing (1-5) | **3.96**  **(3.86, 4.06)** | 3.44  (3.33, 3.55) |
| Recommend Video (1-5) | **4.26**  **(4.17, 4.34)** | 3.75  (3.63, 3.87) |

*Note.* ‘Amount of Information’ variable was rated on a scale from 1 (*much too* little) to 5 (*way too* much). ‘Clarity’, ‘Trustworthy’, ‘Easy to Watch/Understand’, ‘Information Learned’, ‘Understand Patient’s POV’ (understand what it is like to have a colonoscopy from the patient’s point of view), ‘Appealing’, and ‘Recommend Video’ variables were rated on scales from 1 (*strongly disagree*) to 5 (*strongly agree*). ‘Familiarity’ variable was rated on a scale from 1 (*very familiar*) to 5 (*very new*). ‘Reassurance’ variable was rated on a scale from 1 (*very worried*) to 5 (*very reassured*).

Bolded values denote non-overlapping confidence intervals.
