# Supplementary material for: New Patient Education Video on Colonoscopy Preparation: Development and Evaluation Study
Source: JMIR Hum Factors. 2020 Oct 21;7(4):e15353. doi: 10.2196/15353 (PMC7641787; doi:10.2196/15353)
Supplement: Multimedia Appendix 5 [file humanfactors_v7i4e15353_app5.docx]

**Appendix 5. Rating of Characteristics of Current and Revised Video, stratified by low versus high education**

|  | Rating of New Video  Mean (95% CI)  (N=232) | | Rating of Comparator Video  Mean (95% CI)  (N=232) | |
| --- | --- | --- | --- | --- |
|  | Low Education (N=115) | High Education (N=117) | Low Education (N=115) | High Education (N=117) |
| Amount of Information (1-5) | **3.06***  **(3.00, 3.12)** | **3.11**^  **(3.03, 3.20)** | 2.82  (2.72, 2.91) | 2.88  (2.78, 2.98) |
| Clarity (1-5) | **4.40***  **(4.29, 4.51)** | 4.27  (4.12, 4.41) | 3.81  (3.64, 3.98) | 3.97  (3.81, 4.12) |
| Trustworthy (1-5) | **4.29***  **(4.15, 4.42)** | 4.28  (4.15, 4.42) | 3.99  (3.86, 4.12) | 4.11  (3.98, 4.25) |
| Easy to watch/understand (1-5) | **4.37***  **(4.26, 4.49)** | **4.30**^  **(4.18, 4.42)** | 3.95  (3.80, 4.10) | 4.02  (3.86, 4.17) |
| Familiarity (1-5) | 2.51  (2.27, 2.76) | 2.44  (2.19, 2.68) | 2.63  (2.39, 2.86) | 2.57  (2.34, 2.81) |
| Reassure (1-5) | **3.84***  **(3.70, 3.99)** | 3.76  (3.61, 3.91) | 3.50  (3.34, 3.65) | 3.49  (3.34, 3.64) |
| Information Learned (1-5) | **4.13***  **(3.98, 4.28)** | 3.98  (3.85, 4.11) | 3.67  (3.50, 3.84) | 3.81  (3.67, 3.96) |
| Understand Patient’s POV (1-5) | **3.91***  **(3.75, 4.08)** | **3.94**^  **(3.79, 4.09)** | 3.36  (3.17, 3.54) | 3.41  (3.21, 3.61) |
| Appealing (1-5) | **3.93***  **(3.78, 4.09)** | **3.98**^  **(3.84, 4.12)** | 3.39  (3.23, 3.56) | 3.49  (3.33, 3.65) |
| Recommend Video (1-5) | **4.30***  **(4.18, 4.43)** | **4.21**^  **(4.10, 4.33)** | 3.66  (3.49, 3.83) | 3.83  (3.66, 4.00) |

*Note.* Low education = less than 16 years; High education = 16 years and more

Amount of Information was rated on a scale from 1 (*much too little*) to 5 (*way too much*).

Familiarity variable was rated on a scale from 1 (*very familiar*) to 5 (*very new*).

Reassurance was rated on a scale from 1 (*very worried*) to 5 (*very reassured*).

All other variables were rated on scales from 1 (strongly disagree) to 5 (strongly agree).

Understand Patient’s point of view (understand what it is like to have a colonoscopy from the patient’s point of view).

* These bolded values denote non-overlapping confidence intervals in the low education group; Comparison New vs. Comparator video.

^ These bolded values denote non-overlapping confidence intervals in the high education group; Comparison New vs. Comparator video.
