# Supplementary material for: New Patient Education Video on Colonoscopy Preparation: Development and Evaluation Study
Source: JMIR Hum Factors. 2020 Oct 21;7(4):e15353. doi: 10.2196/15353 (PMC7641787; doi:10.2196/15353)
Supplement: Multimedia Appendix 6 [file humanfactors_v7i4e15353_app6.docx]

**Appendix 6**

Correlations Among Evaluation Variables

| Variable | Clarity | Trust | Easy to watch  /understand | Familiarity | Reassurance | Information learned | Patient point of view | Appeal | Recommend |
| --- | --- | --- | --- | --- | --- | --- | --- | --- | --- |
| New video | | | | | | | | | |
| Clarity | 1.0 |  |  |  |  |  |  |  |  |
| Trustworthy | **.54 ^a^** | 1.0 |  |  |  |  |  |  |  |
| Easy to watch  /understand | **.65 ^a^** | **.53 ^a^** | 1.0 |  |  |  |  |  |  |
| Familiarity | .002 | -.01 | -.10 | 1.0 |  |  |  |  |  |
| Reassurance | **.30 ^a^** | **.34 ^a^** | **.34 ^a^** | -.08 | 1.0 |  |  |  |  |
| Information learned | **.41 ^a^** | **.34 ^a^** | **.36 ^a^** | **.18 ^a^** | **.24 ^a^** | 1.0 |  |  |  |
| Patient point of view | **.18 ^a^** | **.18 ^a^** | **.16 ^b^** | -.02 | **.40 ^a^** | **.24 ^a^** | 1.0 |  |  |
| Appeal | **.34 ^a^** | **.34 ^a^** | **.51 ^a^** | -.003 | **.51 ^a^** | **.33 ^a^** | **.40 ^a^** | 1.0 |  |
| Recommend | **.32 ^a^** | **.29 ^a^** | **.46 ^a^** | -.01 | **.54 ^a^** | **.35 ^a^** | **.40 ^a^** | **.71 ^a^** | 1.0 |
| Comparator video | | | | | | | | | |
| Clarity | 1.0 |  |  |  |  |  |  |  |  |
| Trustworthy | **.58 ^a^** | 1.0 |  |  |  |  |  |  |  |
| Easy to watch  /understand | **.63 ^a^** | **.49 ^a^** | 1.0 |  |  |  |  |  |  |
| Familiarity | **.14 ^b^** | -.01 | .04 | 1.0 |  |  |  |  |  |
| Reassurance | **.44 ^a^** | **.38 ^a^** | **.46 ^a^** | .04 | 1.0 |  |  |  |  |
| Information learned | **.55 ^a^** | **.42 ^a^** | **.59 ^a^** | **.20 ^a^** | **.44 ^a^** | 1.0 |  |  |  |
| Patient point of view | **.39 ^a^** | **.31 ^a^** | **.41 ^a^** | .12 | **.44 ^a^** | **.53 ^a^** | 1.0 |  |  |
| Appeal | **.57 ^a^** | **.40 ^a^** | **.64 ^a^** | .07 | **.54 ^a^** | **.57 ^a^** | **.53 ^a^** | 1.0 |  |
| Recommend | **.60 ^a^** | **.49 ^a^** | **.65 ^a^** | .09 | **.56 ^a^** | **.66 ^a^** | **.64 ^a^** | **.74 ^a^** | 1.0 |

*Note.* ^a^ denotes significance at the .01 level (2-tailed).

^b^ denotes significance at the .05 level (2-tailed).
